# Dietary Protein and Amino Acids in Vegetarian Diets—A Review

**DOI:** 10.3390/nu11112661

**Published:** 2019-11-04

**Authors:** François Mariotti, Christopher D. Gardner

**Affiliations:** 1 UMR PNCA, AgroParisTech, INRA, Université Paris-Saclay, 75005 Paris, France; 2 Department of Medicine, Stanford Prevention Research Center, Stanford University Medical School, Stanford, CA 94305, USA; cgardner@stanford.edu

**Keywords:** vegetarian diet, vegan diet, protein, amino acids, adequacy, adults, protein intake, protein requirement

## Abstract

While animal products are rich in protein, the adequacy of dietary protein intake from vegetarian/vegan diets has long been controversial. In this review, we examine the protein and amino acid intakes from vegetarian diets followed by adults in western countries and gather information in terms of adequacy for protein and amino acids requirements, using indirect and direct data to estimate nutritional status. We point out that protein-rich foods, such as traditional legumes, nuts and seeds, are sufficient to achieve full protein adequacy in adults consuming vegetarian/vegan diets, while the question of any amino acid deficiency has been substantially overstated. Our review addresses the adequacy in changes to protein patterns in people newly transitioning to vegetarian diets. We also specifically address this in older adults, where the issues linked to the protein adequacy of vegetarian diets are more complex. This contrasts with the situation in children where there are no specific concerns regarding protein adequacy because of their very high energy requirements compared to those of protein. Given the growing shifts in recommendations from nutrition health professionals for people to transition to more plant-based, whole-food diets, additional scientific evidence-based communications confirming the protein adequacy of vegetarian and vegan diets is warranted.

## 1. Introduction

Globally, human dietary patterns range substantially in the degree of inclusion vs. avoidance of animal-based foods. Vegetarianism refers to the exclusion of meat, fish, seafood and possibly other animal products such as dairy and eggs. In this review, in accordance with standard definitions [1], we will consider as vegetarian diets all those which exclude meat and fish, regardless of whether other animal products such as dairy and/or eggs are also excluded. In the literature; however, vegetarian diets are often lacto-ovo-vegetarian diets (and therefore frequently taken as being synonymous with “vegetarian diets”). Furthermore, the literature frequently reports results on vegan diets (excluding all animal products), which are less common but interesting because they lie at the extreme of the vegetarian spectrum. We acknowledge some degree of caution in this review because the literature on vegetarian diets is inherently complex for many reasons, including (1) a lack of consistency in definitions of vegetarianism, (2) the use of self-reported vegetarianism, (3) heterogeneity within the vegetarian spectrum, (4) errors and uncertainties regarding the nutrient content of vegetarian foods, (5) dietary measurement error regarding protein intake, (6) the representativeness of samples of vegetarians and, of course, (7) the confounding factors present in observational studies which mean that vegetarian diets may not necessarily be causative of the associations with health outcomes observed for vegetarians [1]. We will comment further below on how these problems can limit our understanding of the protein status of vegetarians.

Because vegetarian diets exclude animal flesh, and sometimes all or most other animal-based foods that are rich in protein, the question of whether vegetarian diets can meet protein requirements has long been a controversial topic in the field of nutrition. “Where do you get your protein from?” is a standard question asked of vegetarians, and particularly vegans. Furthermore, in Western countries, reduction of animal protein intake, in particular meat consumption, has recently become more prevalent, for a variety of different reasons [2,3,4]. This recent transitional trend, although apparently not associated with an increase in the number of vegetarians [4], has led to the same kind of questioning about protein adequacy in semi-vegetarian diets, or the so-called flexitarians.

### Protein Intake from Vegetarian Diets

Most articles in the literature have reported a gradient of protein intake from meat eaters to vegans among adults in western countries. Analyzing the results of the EPIC-Oxford study, Sobiecki et al. [5] reported protein intake according to the following gradient: meat-eaters > fish-eaters > lacto-ovo-vegetarians > vegans (Table 1). In the French Nutrinet-Santé cohort, Alles et al. [6] reported almost the same gradient, with similar figures for protein expressed as a percent energy intake (~17.5% for meat-eaters and 13% for vegans; Table 2). The similarity between these figures is quite striking because the studies differed in many characteristics, including their background populations (including dietary culture and population characteristics), how vegetarianism was classified and the proportion of vegetarians in the samples. The studies were similar in the gender ratio of vegetarians (~80–85%of females). In addition, the percent energy intake from protein were also in line with other estimates regarding vegetarians (without any distinctions), such as those reported by the UK women’s cohort study (13.1%) [7] or NHANES (13.5%) [8].

**Figure 1:**
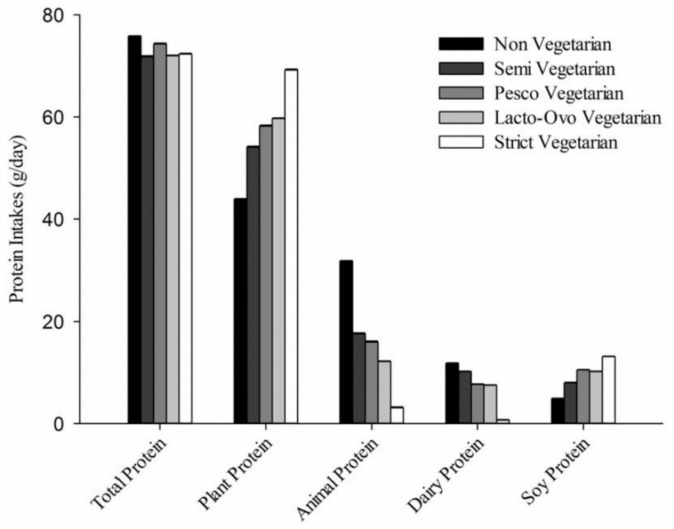
Protein intake (g/day) in the Adventist Health Study 2. From Rizzo and collaborators [9] with permission.

One clear exception from this gradient was reported in the Adventist Studies. In Adventist Health Study 2 (AHS-2), the protein intake of lacto-ovo-vegetarians and vegans were strikingly similar to those of fish-eaters, semi-vegetarians and non-vegetarians [9] (Figure 1). This difference from other populations may be explained by either of two reasons. First, in the Adventist populations [9], the non-vegetarians have a low meat intake and their final protein intake was predominantly plant-based (~60% of protein from plants), in sharp contrast to standard diets in western countries (30% of protein from plants) [10,11]. The median animal protein intake in AHS-2 was reported to be 29 g/day, whereas in France it is 70 g/day [12]. Non-vegetarians in the AHS-2 study have a predominantly plant-based diet, as illustrated by their average intake of fiber being 30 g/day, vs. 22 g/day among non-vegetarians in EPIC-Oxford, 20 g/day in France and 16 g/day in the general US population) [9,13,14,15]. Second, vegetarians, including vegans, had a relatively high protein intake. The median total protein intake in vegans was 71 g, amounting to 14.4% of energy intake [9], which was quite high when compared to other vegan populations, as we will discuss further below. This high plant protein intake in vegans was largely attributable to a classically high intake of protein-rich foods such as legumes in an overall diet that was also rich in whole grains, nuts and seeds.

Finally, we observed that the gradient for protein intake across the vegetarian spectrum is likely due to a high animal protein intake on a background western diet; those transitioning to vegetarian diets from high-animal protein western diets may choose lower protein plant foods compared to those culturally accustomed to more traditional plant-based diets. This point will be discussed in further detail below. 

## 2. Overall Protein Adequacy in Vegetarian Diets

Does the lower protein intake from vegetarian diets mean that this intake is too low to meet protein requirements? In general, protein intakes are high in the overall population. In western countries with the highest level of income, protein intake has risen markedly over the past century, in line with an increasing consumption of animal products, and notably meat [16]. This increase in the contribution of animal products to total energy intake is a central feature of the nutritional transition that has affected western countries in the 20th century and that is ongoing in developing countries [17]. For instance, the total protein intake in Spain rose from 79 g in 1961 to 106 g in 2009, while the proportion of animal proteins increased from 33% to 61%, according to FAO food balance sheets [18]. In most industrialized countries, the protein intake of the general adult population reaches ~100 g/day, i.e., 1.3–1.4 g/kg/day, corresponding to a total energy intake of ~16% [19,20,21]. However, depending on the country, specific region, or gender, total protein intake varies little in the general (meat-eating) population, remaining within the 13–18% range of energy intake [10,20]. In the general adult population in western countries, the average protein intake (~1.3 g/kg/day) is about twice the Estimated Average Requirement (EAR, 0.66 g/kg/day). Therefore, when comparing protein intake in the whole population with a distribution of requirements, it has been concluded that virtually everyone in western populations consumes more than their individual requirement [22]. Against this background, it is expected that, although protein intake is very often lower with a vegetarian diet, it will still be sufficient. There have been only a few studies that have directly reported estimates of protein adequacy in vegetarian populations. As mentioned previously, lacto-ovo-vegetarians (or non-vegan vegetarians) have a protein intake that is ~14% of energy intake in the EPIC-Oxford and Nutrinet studies, which translates to 1.04 g/kg body weight, i.e., 70 g/day. Based on this simple estimate, which is much higher than the Recommended Dietary Allowance (RDA, 0.8 g/kg body weight), few people are expected to have intakes below their requirements, although protein intake varies considerably within populations. The prevalence of protein inadequacy was calculated during the EPIC-Oxford study using the EAR cut-off method, and the authors found figures of 10% in men and 6% in women (compared to 3% in male meat-eaters and 1% in female meat-eaters). Taken together, although there are some uncertainties regarding these estimates, they nevertheless suggest that a modestly higher proportion of lacto-ovo-vegetarians than meat-eaters could have protein intakes that do not meet their individual requirements.

Could the level of inadequacy be higher in vegans, who have lower intakes? To address this question, we first obtained estimates of protein intakes from the literature among subsamples of vegans, including the three largest samples (EPIC-Oxford, Nutrinet and AHS-2); in order to expand this overview, data from two smaller studies were also included (Table 3). Most of the studies reported an average protein intake of ~13–14% energy, which is clearly higher than the 10% threshold considered as the lower bound of acceptable intake [23]. Likewise, average intakes ranged from 62 g/day to 82 g/day, well above the 50 g which is often taken as a very approximate reference value. Beyond simple comparisons between the overall means, two studies provided comparisons of population samples with reference values. In the French Nutrinet-Santé sample, 27% of vegans had values lower than 10% energy. However, a comparison with this reference as % of energy only remains an indirect assessment. In the EPIC-Oxford sample, the proportions of vegans with a protein intake lower than their requirement (based on the EAR cut-off method) were 16.5% of men and 8.1% of women. We estimated the confidence intervals using sample sizes and prevalence estimates as [12.1; 20.9] for men and [5.8; 10.4] for women. These survey results tend to indicate that a small fraction of vegans may have an insufficient protein intake, and this phenomenon may be obscured by a much higher and very sufficient intake in the overall population. In other words, evaluating the nutritional status of vegans is hampered by the challenges of both accurately capturing highly variable intakes within the vegan population, and being able to accurately differentiate individuals with high vs. low individual protein requirements. In this sample, the average intake was 0.99 g/kg body weight, which is substantially higher than the RDA of 0.83 (the value that meets or exceeds the requirement of 97.5% of the population), and yet 16.5% males and 8.1% females have an intake below the EAR of 0.66 g/kg, which is admittedly low.

In this regard, the results of the AHS study were once again at variance with the findings reported in other studies for other populations. There was no reported population-based estimate of the prevalence of protein inadequacy in this population, but clues were given by the reported 5th percentile values for the vegan population, where protein intakes were 54 g/day in non-vegetarians, 53 g/day in lacto-ovo-vegetarians and 52 g/day in vegans. In the latter, protein intake as a percentage of energy was 10.1% at the 5th percentile. It therefore appears that even the lowest intakes were generally higher than the reference value, from which it can be concluded that their protein intake was likely adequate, except for just a small percentage of the vegan population in this survey, as usually found in the general population. Note, this finding may not be specifically an issue of protein deficiency; it could also be a consequence of energy intake deficiency. Why these results differed from those found in the EPIC-Oxford vegans remains unclear. As we have already noted, the average protein intake in vegans was higher in AHS-2 than in the EPIC-Oxford study (Table 3) so this may mean that the entire sample had an overall higher intake. It may also mean that the AHS-2 vegans are more homogeneous, with less variance in their protein intake so that very few people had a very low protein intake. This could be expected because the total AHS-2 population (i.e., including vegetarians and non-vegetarians) is already more homogeneous in term of its dietary pattern than the EPIC-oxford population [13], which was also consistent with the fact that Adventists live in communities with stronger cultural bounds. 

### Limitations when Estimating Protein Inadequacy from Dietary Intake Surveys

All estimations of the prevalence of protein inadequacy should be considered with caution, given the well-known limitations when estimating nutrient intakes from dietary surveys. Such estimates are affected by uncertainties and biases that tend to accumulate (e.g., errors in frequencies, portion sizes, nutrient composition, etc.). For protein intake, standard methods (repeated 24 h recalls or records, or food frequency questionnaires) offer good accuracy when classifying subjects into categories of relatively higher vs. lower intakes, but absolute quantitative precision remains low. When making quantitative estimates of individual foods or nutrients these uncertainties are particularly important at the lower and upper ends of the distribution; problems with accurately estimating usual intakes suggest that distribution estimates tend to be larger than reality. Errors in the extreme ranges of protein intake (such as the lowest level) are difficult to quantify or characterize because naturally few of these data are available and the validation methods themselves are not free of errors [27,28,29,30]. When estimating the prevalence of inadequacy using standard methods, uncertainties tend to overestimate the proportion of individuals with insufficient intakes [31,32]. This problem is known to be less marked for protein (which is largely and frequently consumed) than for other nutrients [32], but the uncertainties may be greater in vegans because their protein intake is distributed over a larger number of contributors and may therefore be estimated with a lower degree of precision from a food frequency questionnaire. It would be interesting to examine the combined distribution of energy and protein intakes in studies that have considered vegetarian diets; however, detailed data on the distribution of protein and energy intake are not usually available. Many studies, even when excluding clear under-reporters, contain data where a fraction of the population display very low estimated energy intakes, and these surely include underestimates of actual energy intake that cannot be differentiated from true energy intake estimates. Such errors are expected to be associated with underestimated protein intakes, which then tend to overestimate protein inadequacy. For instance, the 5th percentile of energy intake in vegans in the AHS-2 study was 910 kcal/d, which is an energy intake that would be very insufficient for weight maintenance, even for females with small stature and little physical activity. In such populations, the authors corrected and standardized nutrient intake estimates to 2000 kcal/d in order to prevent this error from becoming an error in the risk of insufficient nutrient intake. Please note that this correction is probably insufficient inasmuch average energy intake, as determined by doubly labelled water studies are presumably quite higher [33]. Generally speaking, under-reporting and imperfect methods to manage under-reporting have been shown to affect estimates of nutrient intake, with overestimation of the prevalence of protein inadequacy [34]. 

Finally, from the dietary data at hand we can deduce two complementary pieces of information. First, some vegans in the general population may be at risk of having a protein intake that is lower than the estimated requirement, although more precise data need to be collected to accurately assess the proportion of the population in this lower end of the distribution. In this context, low protein intake may also be primarily an energy intake deficiency. Second, most vegetarians (including vegans) have an adequate protein intake overall and some of the individuals in this population have notably high levels of intake, which is in line with other analyses [10,25,35]. The heterogeneity of these populations is indeed a key finding. Future studies that address the factors that explain this low (or high) protein intake in vegetarians are warranted.

## 3. Amino Acid Adequacy in Vegetarian Diets

It is commonly, although mistakenly, thought that the amino acid intake may be inadequate in vegetarian diets. As we and others have argued, the amounts and proportions of amino acids consumed by vegetarians and vegans are typically more than sufficient to meet and exceed individual daily requirements, provided a reasonable variety of foods are consumed and energy intake needs are being met. The claim that certain plant foods are “missing” specific amino acids is demonstrably false. All plant foods contain all 20 amino acids, including the 9 indispensable amino acids [33]. Importantly, rather than “missing” indispensable amino acids, a more accurate statement would be that the amino acid distribution profile is less optimal in plant foods than in animal foods. Lysine is present in much lower than optimal proportions for human needs in grains, and similarly the sulfur containing amino acids (methionine and cysteine) are proportionally very slightly lower in legumes than would be optimal for human needs. This would be important for someone who ate only rice or only beans, for sustenance, every day. This classic implementation of a protein quality assessment framework focusing on isolated single proteins remains an erroneous approach in practice [36,37]. The terms “complete” and “incomplete” are misleading [33,38]. In developed countries, plant proteins are mixed, especially in vegetarian diets, and total intake of protein tends to greatly exceed requirement. This results in intakes of all 20 amino acids that are more than sufficient to cover requirements. In the EPIC-Oxford study, amino acid intakes were estimated in both meat-eaters and vegetarians [24]. For the lacto-ovo-vegetarian and vegans assessed, based on an average body weight of 65 kg, we calculated that lysine intakes were 58 and 43 mg/kg, respectively, largely higher than the 30 mg/kg estimated average requirement [39]. An insufficient intake of lysine is not therefore expected in these populations. Granted, inadequate lysine could be more likely in vegans, where a very high proportion of their protein intake comes from cereals only. However, even when eating a plant-based diet of limited variety, significant amounts of total protein can be achieved from a high intake of low-protein foods such as vegetables and fruits [11].

Another factor to consider is differential rates of protein digestibility that impact amino acid availability, often considered as being poorer for plant proteins. This remains a matter of debate. There is very little evidence at present regarding a marked difference in protein digestibility in humans. The more precise data collected so far in humans, assessing real (specific) oro-ileal nitrogen digestibility, has shown that the differences in the digestibility between plant and animal protein sources are only a few percent, contrary to historical findings in rats or determinations using less precise methods in humans [37]. For soy protein isolate, pea protein flour or isolate, wheat flour and lupine flour, the figures were 89–92%, similar to those found for eggs (91%) or meat (90–94%), and slightly lower than those reported for milk protein (95%). It is important to note that most of the plant proteins studied came from raw, untreated (unheated, or minimally heated) sources, and some were ingested in complex food matrices such as (unheated) flour [37], i.e., in the worst conditions for plant protein because of the presence of trypsin inhibitors and the poor enzyme accessibility of some native proteins. While further research may be warranted to explore possible variations in the bioavailability of some specific amino acids, the body of evidence so far does not show a difference large enough to result in risk of insufficient amino acid absorption for vegetarian and plant-based diets

Finally, if the proportions of specific amino acid intakes from vegetarian diets are inadequate for meeting total protein requirements at the reported RDA levels of 0.8 g/kg body weight, then there would need to be a separate, higher, total protein RDA for vegetarians. This case is, however, at variance with the results of the data that have been used to directly estimate protein requirements. In a meta-regression of nitrogen balance studies in humans, Rand and colleagues [40] examined the protein sources in three separate groups: animal, vegetable, and mixed. They found no differences in the slope or the intercept for nitrogen balance in these three subgroups, suggesting that total protein requirement is similar with plant-based or animal-based diets [39,40]. When interpreting this analysis, however, it is important to note that the “vegetable” diets included mixtures of plant proteins (cereals and legumes) or good quality soy protein; there were no rice-only, or bean-only diets. Therefore, the evidence suggests a similar total protein requirement to those following western vegetarian diets in general [41] or diets rich in both cereals and legumes [42]. Overall, when diets are at least slightly varied, suggestions that vegetarians to be sure to achieve a higher total protein intake than the RDA, or to pay strict attention to choosing plant foods with complementary amino acid patterns are simply over-precautious. 

## 4. There Is No Evidence of Protein Deficiency among Vegetarians in Western Countries

Because the indirect assessment of protein intake adequacy remains difficult, it is logical to consider the direct assessment of nutritional status, and thus question the existence of biological or physiological markers for an insufficient protein intake in vegetarians. The effects of a vegetarian diet on protein metabolism and status have been investigated in a small number of studies. One example of evidence for a difference in protein metabolism depending on diet type was provided in the seminal controlled trial performed by Caso et al. [43]. The authors measured the albumin synthesis rate in healthy humans after 10 days of consuming a diet containing 78 g of protein which either came mostly from animal protein (74% animal) or plant protein (67% plant), which they considered as “vegetarian”. They found that the vegetarian diet reduced albumin synthesis by 13 ± 13%. They also showed that this decrease was alleviated when the protein intake increased to 96 g in the vegetarian diet. Such a decrease in albumin synthesis could have been expected to result in lower plasma albumin concentration. However, the authors found a very moderate decrease (2.5%). Notably, this could have been explained by the fact that the diet was given for a period that was too short. Nonetheless, other authors did not find any difference in albumin concentration when comparing vegetarian and non-vegetarian populations [44] and indeed, in one study higher concentrations (5%) were reported in vegans [45]. The reason for a higher albumin concentration in vegans remains unknown, although one possibility may be a different plasma albumin turnover rate, while another may be a long-term reallocation of the synthesis of exported proteins by the liver, with vegetarians displaying lower levels of pro-inflammatory plasma protein [46,47]. Historical reports on this issue can be traced back to the 1950s. In 1950, Mirone reported that a group of vegetarians consuming 50 g total protein plus ~6 g animal protein was in apparent good health, as indicated by their normal hematology and blood chemistry markers (including serum albumin) and this was taken as an early indication that a plant protein combination could replace animal protein [48,49]. 

Few studies have analyzed postprandial anabolism in individuals receiving a plant protein-based diet of mixed origin or an animal protein-based diet. Most of the data have been obtained on isolated proteins, such as wheat and soy, and these studies reported a lower stimulation of whole-body retention or muscle anabolism as compared to animal protein [50,51]. It has notably been shown for many years that the utilization of a single isolated plant protein for whole-body protein anabolism during the postprandial period is better than what can be predicted from their amino acid composition, which is proportionally low in one amino acid (e.g., lysine in wheat) when compared to the reference pattern-based of amino acid requirement [52,53]. In line with earlier evidence [54], these findings can be taken as an illustration of the fact that protein synthesis in adults is not very sensitive postprandially to specific amino acid composition with respect to the daily amino acid requirement. Indeed, there is no true reservoir of certain specific amino acids that could be used between meals to smooth variable intakes of each amino acid. Such buffering operates in a limited manner, and in part via total free amino acid pools. It has been suggested that the relatively large free lysine pool (as compared to leucine and other indispensable amino acids) enables lysine recycling during diurnal cycling, which in turn enables the conservation of lysine between meals, at least to some extent [53]. This is in line with an earlier study in rats which showed that the utilization of a lysine supplement added to a deficient diet over 24 h was similar to that of lysine given 12 h after feeding with a deficient diet [55]. Evidence that this would operate for more than 24 h remains scarce, but such a mechanism would explain a lower demand for lysine for maintenance at lower intake of lysine [56]. It has also been suggested that variations in cysteine intake may be buffered because of its involvement in other pathways, and notably the existence of a large pool of cysteine in the form of glutathione [52]. By contrast, tryptophan is known to be tightly regulated and have a small pool that is rapidly turned over, thus impairing its potential to buffer variations in intake [55]. However, tryptophan is present in similar quantities in plant and animal proteins and in sufficiently high amounts when compared to the requirement [57]. Therefore, there is ultimately no evidence yet that isolated single plant proteins (which can be low in lysine only and very occasionally in methionine) need to be supplemented with other proteins in the same meal, and a reasonable variety of sources over the course of the day appears to be appropriate [38,54]. Mixing complementary protein sources within the same meal may simply be a practical way to secure long-term adequacy if and when the total protein intake is low.

When studying plant protein mixes in experimental diets used to mimic vegetarian diets, some investigators have reported trends toward differences in protein metabolism (such as a lower postprandial decrease in protein breakdown when vegetarian diets are consumed). However, there has been no evidence for any impact on nitrogen balance, therefore making it difficult to speculate whether these effects of the protein sources have any biological implications [58]. Evidence for differences in lean or muscle mass remains anecdotal, and has all come from small cross-sectional studies [59], and the clinical significance of these potential differences is uncertain.

The overall conclusion from this review of the literature is that mixing plant protein sources, as is typically done in vegetarian diets, should address any issues with inadequacy that have been found when studying single sources [50] so it is, therefore, not surprising that there is no evidence of protein deficiency in vegetarian populations in western countries. 

We conclude that protein intakes from vegetarian diets are sufficient, except possibly in a fraction of vegetarians who are not consuming sufficient energy intake, or who habitually for some reason avoid protein-rich plant sources such as legumes, nuts and seeds, or protein-rich analogs. Amino acid intakes are sufficient and lysine intake might only be limiting in vegan individuals who have a low protein intake when basing their diet on a very limited and monotonous pattern where the protein intake would only come from, for example, grains alone—an unrealistic situation in developed countries.

## 5. Plant Protein Sources in Classic Vegetarian Diets and Lessons Regarding Future Trends towards Vegetarian Diets

Classic vegetarian diets involve the use of standard protein-rich food products. In that sense, classic vegetarian diets in industrialized countries can be seen in a historical perspective, sharing some common ground with the principal dishes that enabled populations to thrive. Legumes, as a complement to cereals, are probably one of the oldest features of “cultural nutrition”, and they remained as part of vegetarian diets in western countries while being abandoned by the general population in favor of meat during the 20th century. Numerous traditional dishes that are staples in many cultures throughout the world combine cereals with legumes or dairy, such as wheat and chickpeas (in couscous), diverse breads or pastas with cheese, or the famous rice and beans. Legumes have been central to many cultures all over the world. They are known to complement cereals at low levels of protein intake, but of more importance is the fact that legumes are rich in protein, so they are an important component in classic vegetarian diets. Industry has also developed plant analogs to animal-based foods that have long been used by vegetarian communities, and particularly analogs for meats and delicatessen products for vegetarians (e.g., vegetarian patties) or dairy analogs for vegans (soy-based products such as tofu), and they are usually comparable in terms of their protein content. Finally, classic products such as legumes, and more recent products such as analogs, have long offered adequate sources of protein for consumption by vegetarians. 

**Figure 2:**
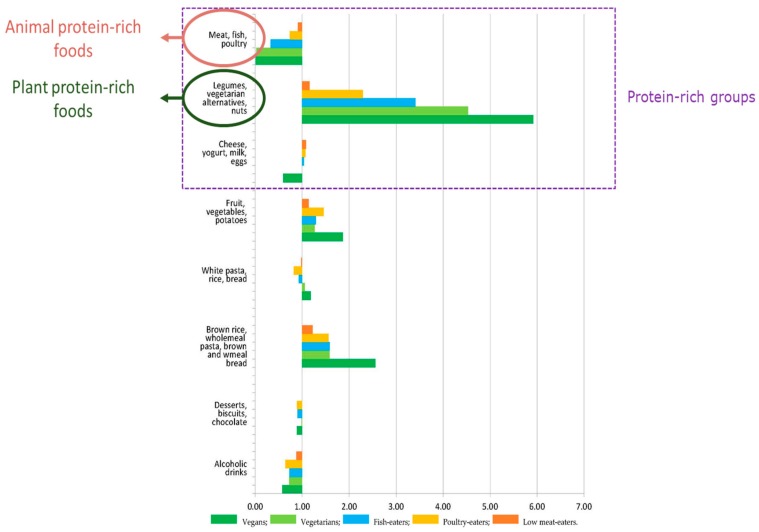
Relative consumption of food groups (g) in low meat-eaters, poultry-eaters, fish-eaters, vegetarian, and vegan men compared to regular meat-eaters in the EPIC-Oxford study. The mean consumption relative to regular meat-eaters (1.00) is shown for each food group after adjustment for age. Circled are intakes of animal or plant-protein rich food groups. Adapted from [61].

In the Nutrinet study, while meat-eaters consumed 47.1 g meat and 11.5 g legumes, vegans consumed 73 g legumes and 63 g of textured soy protein and vegetarian patties, which was in addition to the intake of grain products. An earlier report on vegan Adventists had also revealed high intakes of legumes and analogs [45], and a more recent analysis of AHS-2 confirmed that vegans had very high intakes of legumes, soy-based foods and meat analogs, as well as nuts and seeds [60]. In this Adventist population, the intake of these protein-rich foods by vegans was approximately twice that of non-vegetarians, but again the latter had a prudent diet with relatively high intakes of such plant protein-rich foods when compared to a general non-vegetarian population. By contrast, when compared to a more general population, it was reported that vegans in the UK Biobank study had intakes of legumes, vegetarian alternatives and nuts that were ~6 fold higher than those of regular meat-eaters [61]. There was also an increase in the consumption of foods rich in plant proteins, mirroring the reduction in animal protein foods, with steps corresponding to dietary types along a graded spectrum including vegetarian diets (Figure 2). Similar trends were found in the EPIC-Oxford study [62].

Vegetarian diets are varied in western countries. As discussed, protein intake differs within the same sample of vegetarians, according to their different consumption patterns [63]. It would be interesting if future studies could analyze precisely whether the subset of vegetarians with the lowest protein intakes do avoid or limit the most protein-rich plant foods such as legumes or analogs for some reason, and whether these foods are necessary to obtain protein at individual requirement levels for those with the lowest protein intakes. The great unknown is how the transition toward more plant and less animal protein will occur in countries or communities where there is little background culture with respect to vegetarian foods. It is possible that the transition in certain newly vegan individuals (e.g., those transitioning primarily for the purpose of promoting animal rights and welfare) [4] could involve preferences for particularly low-protein plant-based foods (e.g., raw food diet without meat analogs and also without legumes) thus potentially increasing the risk that the total protein intake could be inadequate. Beyond the scope of this review also lies the question of the overall nutritional profile (e.g., vitamins, minerals) of the plant sources consumed as an alternative to animal sources.

**Figure 3:**
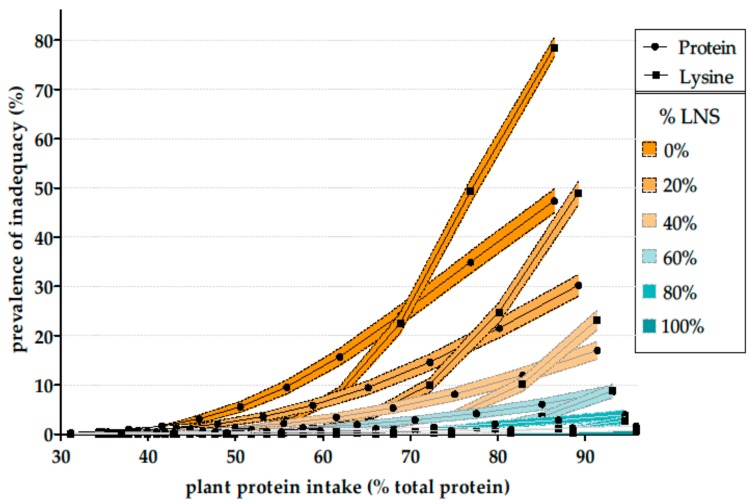
Prevalence of protein and lysine adequacy (% of the INCA2 study population, *n* = 1678) in simulations of a reduction in animal protein intake by gradually balancing it against the same amount of energy from a replacement combination composed of plant foods already consumed by individuals and a mixture of legumes, nuts and seeds. For example, the “40%” curves show the protein and lysine inadequacy when substituting animal protein with a combination of 40% of protein from legumes, nuts and seeds, and 60% of plant protein from foods already consumed by the individuals. The filled area represents the 95% confidence interval. LNS: legumes, nuts and seeds. Reproduced with permission from the authors [57].

From the above discussion, we can conclude that protein intake of vegetarians or vegans is not primarily a question of specific amino acid distributions but more likely one of the total protein intake. When modeling the transition toward plant protein in a general population, we came to the same conclusion. Indeed, when we modeled the isoenergetic replacement of animal protein foods with different types of plant protein foods and mixes in the French adult population, we found that some substitution scenarios that consisted of replacing animal protein with the current cereal-based plant protein intake resulted primarily in increasing the risk of an insufficient intake of total protein rather than of individual amino acids such as lysine—the risk of inadequacy of the latter always lagging well behind that of the former [57]. This could be explained by the low level of protein (over energy) in cereal-based foods as they are currently consumed in the general population of western countries. Accordingly, we found that when replacing animal protein with a mix of protein-rich plant foods (namely legumes, nuts and seeds), a transition toward 100% plant protein could be considered to involve virtually no risk of an insufficient intake of protein, including amino acids such as lysine (Figure 3).

## 6. The Case of Specific Issues in Specific Populations

Data on protein and amino acid intakes from vegetarian diets remain scarce in the general adult population, and are insufficient to assess the status of more specific populations. However, as we will discuss here, there are good reasons to consider the case of older people as being more complicated than that of adults, while the situation in children is even simpler. We will briefly present these cases and the background limitations as we know them, as well as possible lessons regarding the shift towards high plant protein diets.

### 6.1. Older People

First, in older adults, it remains a matter of debate whether the protein requirement is indeed higher than that of younger adults, or is only higher in the frail elderly [64]. Details on this controversy and the limits of our current knowledge can be found elsewhere [65]. In France, the RDA has been set at 1 g/kg [22], which is in line with recent recommendations for working groups [66], while other agencies have chosen not to endorse a higher RDA [39], in line with other reports [67]. In France, for the generally non-vegetarian population of older people, it has been estimated that even with such a higher estimated protein requirement, very few people would be at risk of insufficient intake (3–5% of the older (>65 years) population). However, this risk would increase in vegetarians, and particularly in vegans, if extrapolating using the classic gradient of protein intake described above. The risk would also increase in even older adults [68], mostly because physical activity tends to decrease with age in the same way as energy intake, hence the risk of a low protein intake from diets that are not relatively rich in protein (as protein:energy). Therefore, some elderly people eating a low animal protein diet may have a marginal protein intake when compared to their requirement, and, as predicted by the protein:energy approach, this is probably the case among elderly vegan women of small stature who have an inactive lifestyle [35,39]. Protein intake is usually lower in the vulnerable elderly, such as those who are institutionalized [69] and the protein requirement may be even higher in the frail elderly who are at risk of malnutrition because of acute or chronic illness [66].

Furthermore, there is considerable evidence that older people are resistant to postprandial anabolic stimulation by meal protein, and that this resistance can be overcome by supplying daily protein in the form of protein-rich meals [70,71]. A higher level of postprandial anabolism was shown in older people (but not younger adults) following a single large protein meal versus several smaller ones [72,73,74]. It is usually considered that meals containing ~30 g protein are necessary to pass the “anabolic threshold” and optimize postprandial anabolism [70,75,76], and therefore favor lean mass and strength [77]. Recent data from the NuAge cohort have however challenged this viewpoint, showing that a more even protein intake distribution across meals was associated with a higher lean mass and appendicular lean mass, and also muscle strength, in older men and women [78,79]; estimated meal protein in the more even distribution was typically lower than 30 g, especially in women. Indispensable amino acids, and particularly branched-chain amino acids (BCAA), are considered to be key in eliciting this anabolic response in the postprandial state, so a threshold (3 g) for peak anabolism has also been proposed for meal leucine [80], which triggers a signal for the anabolic utilization of the bulk of amino acids [81,82]. These metabolic aspects have implications for protein nutrition in the elderly beyond their basic requirement for amino acids and our consideration of the potential of protein from different sources with respect to protein nutrition. To achieve optimum benefits in the elderly, a diet should not just contain sufficient BCAA (when compared to what is required for a high PDCAAS), but the protein-rich food sources in a meal should be rich in BCAA. Indeed, most sources of plant protein are similarly rich in leucine when compared to animal protein (i.e., an average of 8 g/100 g, ranging from 6 to 14 g in a set of 18 plant proteins selected from the USDA database versus an average of 9.5 g/100 g, ranging from 8 to12 g in a set of six animal proteins) [37]. In France, where 31% of protein in the diet comes from plants, plant protein supplies 26% of dietary leucine [57]. Except in the case of highly specific strategies using specific plant proteins that are very rich in leucine (e.g., maize and alfalfa), mixing different proteins is not expected to be necessary when preparing meals with a sufficient leucine content [37,83]. At present, protein-rich meals in the elderly are clearly associated with a higher intake of animal protein [84,85]. Strategies regarding the optimal utilization of plant protein in the diets of older vegetarians remain a subject of interest for future research [50,86].

It should, however, be recognized that although they are expected, the long-term effects of such a postprandial modulation of protein anabolism have not been documented. We still do not know how protein intake from vegetarian diets may influence long term markers of muscle mass and function, fitness and ultimately the risk of sarcopenia, and how those processes might interact with other nutritional and lifestyle factors [87,88]. For instance, only a few longitudinal observational studies have investigated the association of total, animal and plant protein intake in older persons with lean mass changes [89,90,91], with contrasting findings. In relatively healthy elderly subjects in the US, aged ~73 years at baseline and followed for 5 years, Verreijen et al. recently reported that total, plant and animal protein intakes were not associated with changes in muscle mass [92].

### 6.2. Children

The case in children markedly contrasts with the questions at issue in older people. In children, the reference value for protein intake has been based on a factorial approach, which consists in combining estimates of standard maintenance requirements for specific body weight, with an additional component to account for specific requirements due to protein deposition during growth. However, as has long been established [93], even in very young children the protein requirement is mainly driven by the demand for maintenance rather than growth. In a 3-year-old child, the additional component for growth is only ~10% of the requirement for maintenance [39]. For instance, the RDA for children aged 1, 2, 3 and 10 years are 1.14, 0.97, 0.90 and 0.91 g/kg/day, respectively [39]. By contrast, energy requirements for children are very high. Accordingly, when expressing the protein reference intake as a percent of dietary energy, the value gradually and dramatically falls with decreasing age from 18-year-old adults backwards. This reference intake is about 5–6% of energy intake as protein for children aged 3–9 years, and about 8–9% in older children (10–13 years) [94].

The protein intake of children in western countries is very high when compared to these references. In Europe, the average protein intake in 4–6-year-old children is ~55 g/day [20]. The lowest intake seen among European children of that age (5th percentile) is 32 g/day [95], which is still more than twice the RDA. Although a tolerable upper level of intake has not yet been set, French Agency [22] used the upper threshold to characterize protein intake and estimated that most children, especially in the younger age groups, had intakes qualified as “high” or even “very high” (i.e., exceeding 3.5 g/kg/day).

In vegetarian children, these reference intakes for protein as a percent of diet energy can be used to draw conclusions as to the risk of protein inadequacy in young children: protein intakes lower than 6% energy are practically impossible to achieve, including under the most restrictive vegan diets (unless fully abnormal and caricatured), so that the protein adequacy of the diet is fully secured for children aged 3–9 years as soon as they consume sufficient energy. This will also hold true for younger children (1–3 years), because their higher protein requirement (14% higher) as compared with 3–9-year old children is matched by their higher energy requirement (14% higher) [96]. Likewise, in children in general, the indispensable amino acids requirements are higher than in adults but approximately proportional to the protein requirement; so the question of the amino acid patterns in plant-based diets are basically the same for children as for adults, i.e., concerns have been overstated, as discussed above. In infants where energy, protein and amino acid requirements are high, protein requirement are primarily met by intakes of human milk and/or commercial infant formula and it is obvious that infants must be provided with those very specific food, otherwise they will be malnourished [97]. Complementary feeding in 6–12-month-old infants should also be rich enough in protein so that it reaches 10% energy and in vegetarian infants this would be secured by continuing the intake of breast milk or formula [97].

## 7. Nutrient Adequacy of Vegetarian Diets

This review has not analyzed the relationship between protein intake from vegetarian diets and the nutrient adequacy of the diet. In both vegetarian diets and general western diets, choices of types or categories of protein-rich foods shape overall nutrient intake and therefore overall nutrient adequacy [12,98,99,100]. Another topic not addressed in this review is the relationship with long-term health beyond the satisfaction of protein requirements for lean body mass and related functions. There are, however, numerous findings in the recent literature which indicate that a higher plant protein intake and lower intake of some animal proteins likely contribute to the lower risk of disease associated with vegetarian diets [101,102,103,104]. We recently reviewed this evidence [105] and argued that this potential benefit of a higher plant protein intake probably stems from the cluster of nutrient intakes that are closely associated with plant protein [11,106] and also from a different pattern of amino acid intake, with higher contributions from non-indispensable amino acids (such as arginine and cysteine) and lower contributions from indispensable amino acids such as BCAA. In a recent study, we found that pattern of amino acids intake that are contributed by indispensable amino acids intake (vs. those contributed by so-called non-indispensable amino acids) are indeed strongly associated with cardiovascular mortality [107]. This scenario warrants further research to reevaluate the traditionally stated, and probably overrated, importance of indispensable amino acids when plant-based diets are considered in the context of health and disease in the populations of industrialized countries [105]. Indeed, the question of nutritional quality for protein rich foods has been long restricted to the ability to provide indispensable amino acids, whereas plant protein sources are considered critical to overall diet quality and long-term health, calling for a modernized definition of protein quality that incorporates the quality of health and environmental outcomes associated with specific food sources of protein [100].

## 8. Conclusions

Although uncertainties remain regarding protein requirements, the data in adult vegetarians (depending on the methods and criteria used) indicate that classic vegetarian diets supply more than adequate protein and amino acids. In a fraction of vegans, there might be a modest risk of insufficient intake, and further data are needed to assess the actual dietary pattern of people who report dietary intake corresponding to a low intake of protein and energy. An insufficient protein intake from vegetarian diets may occur if the diet does not include protein-rich foods such as legumes (the most traditional source) and nuts and seeds, or any protein analogs of animal foods, the availability of which is increasing along with the proportion of people shifting their protein intake towards more plant protein sources. Beyond vegetarian diets per se, this review has shown that protein foods and overall protein patterns are important characteristics of a diet that is more based on plants than the classic animal-based diets seen in western countries. If a diet has at least a modest amount of variability (which is the case in economically developed countries) there are no issues regarding sufficient intakes of any individual indispensable amino acids from vegetarian diets, including lysine. There is no evidence of any adverse physiological effects of the modestly lower protein intake seen in adults consuming vegetarian diets. In older people, it could be argued that some vegetarian diets might supply insufficient protein to ensure a long-term nitrogen balance and that some vegetarian meals may provide insufficient protein and leucine to favor postprandial anabolism; an issue that warrants further investigation. However, any evidence for a functional impact and higher final risk (of sarcopenia) in the healthy elderly is currently lacking. By contrast, children who are consuming enough energy to cover their requirements for growth should automatically obtain sufficient protein intake from vegetarian diets. We recommend that further study on protein in vegetarian diets shift away from unnecessary questions about protein adequacy, to a comparison of overall nutrition quality and implications for long-term health with plant-based protein-rich foods vs. animal-based protein rich foods.

## Figures and Tables

**Table 1:** Protein intake of vegetarians compared to meat-eaters in the EPIC-Oxford study, classified according to answers to questions on whether those involved ate any meat, fish, eggs, and dairy products. Data from Sobiecki et al. [5].

	Meat-Eaters	Fish-Eaters	Lacto-ovo-Vegetarians	Vegans
*n* (%)	18,244 (60)	4531 (15)	6673 (22)	803 (3)
Energy (kcal)	2091	2030	2002	1944
% Energy from protein	17.2	15.5	14.0	13.1
Protein (g/kg of body weight) ^1^	1.28	1.17	1.04	0.99
Protein (g) ^2^	90	79	70	64
Body weight (kg) ^2^	70	67	67	64

^1^ Based on a subsample of 29,028 individuals with information on body weight; ^2^ As calculated by ourselves.

**Table 2:** Protein intake of vegetarians compared to meat-eaters in the Nutrinet-Santé Study, based on declarations about being a vegetarian (i.e., not eating meat but eating other animal products) or a vegan (not eating any meat, fish, eggs or dairy). Data from Alles et al. [6].

	Meat-Eaters	Neither Meat-Eaters nor Vegan	Vegans
*n* (%)	90,664 (96.6)	2370 (2.5)	789 (0.8)
Energy (kcal)	1899	1814	1877
% Energy from protein	17.6	14.2	12.8
Protein (g) ^1^	84	64	60

^1^ As calculated by ourselves.

**Table 3:** Average protein intake of vegans based on different samples in the literature.

Study	Protein Intake			Vegans (*n*)	Method	Ref.
	%E	g	g/kg bw			
EPIC-Oxford (UK)	13.1	64 ^1^	0.99	803	FFQ ^2^	[5,24]
Nutrinet (France)	12.8	62		789	Multiple 24-h R	[6]
AHS-2 (North America)	14.1	71		5694	FFQ	[9]
A Belgian study	14	82		102	FFQ	[25]
A Danish Survey	11.1 ^1^	67		70	4-d weighted Record	[26]
Recommended Dietary Allowance (RDA)	>10 (approx.)	50 (approx.)	0.83 (exactly)			

^1^ As calculated by ourselves; ^2^ FFQ: Food Frequency Questionnaire; 24-h R: 24-h records.
